# Association Between Age and Severity at Disability Onset and All-Cause Mortality: Longitudinal Observational Study From the Health and Retirement Study

**DOI:** 10.2196/73254

**Published:** 2025-10-03

**Authors:** Anying Bai, Cuie Liu, Yu Jiang, Weihao Xu, Jian Cao

**Affiliations:** 1 School of Population Medicine and Public Health, Chinese Academy of Medical Sciences and Peking Union Medical College, Beijing, China; 2 The 4th Healthcare Department of the Second Medical Center, Chinese PLA General Hospital, National Clinical Research Center for Geriatric Diseases, No. 28 Fuxing Road, Haidian District, Beijing, 100853, China, +86-13910730005; 3 School of Health Policy and Management, Chinese Academy of Medical Science and Peking Union Medical College, Beijing, China; 4 Department of Geriatrics, Guangdong Provincial Geriatrics Institute, Guangdong Provincial People's Hospital, Guangdong Academy of Medical Sciences, Southern Medical University, Guangzhou, China

**Keywords:** disability onset, mortality, older population, severity, matched control

## Abstract

**Background:**

Disability is a global public health challenge, with its prevalence increasing, particularly among older adults, and it exerts a profound impact on both health outcomes and mortality rates.

**Objective:**

This study investigates the associations between age at disability onset, severity at disability onset, and all-cause mortality in community-dwelling adults.

**Methods:**

We analyzed data from waves 10 to 16 (2010‐2023) of the Health and Retirement Study, a nationally representative longitudinal survey of US adults aged ≥51 years. Participants without disabilities in activities of daily living (ADLs) or instrumental activities of daily living (IADLs) from the Health and Retirement Study were followed biennially until December 31, 2023. During the follow-up period, 4500 participants developed ADL disability and 4260 developed IADL disability. For each case participant, a control participant matched for age (+1 to −1 y) and sex was randomly selected. Multivariable Cox proportional hazards models were used to assess hazard ratios (HRs) for all-cause mortality among participants with new-onset disabilities, stratified by age groups and severity at disability onset.

**Results:**

Over a median follow-up duration of 8.58 years, 1709 (37.98%) deaths occurred in the ADL group and 1832 (43%) deaths occurred in the IADL group. Individuals who developed ADL disability before the age of 55 years exhibited the highest all-cause mortality risk compared to matched controls (HR 3.12, 95% CI 1.85‐5.26), which further increased with severe disability (HR 4.07, 95% CI 2.03‐8.19). The mortality risk was inversely associated with age at onset. A parallel trend was identified in the IADL cohort. Notably, men demonstrated a significantly elevated mortality risk compared to women, emphasizing the need for gender-specific interventions.

**Conclusions:**

Early and severe disability onset significantly increases mortality risk, with men experiencing a disproportionately higher risk. Preventive strategies aimed at addressing early-onset and severe disability, with consideration of gender differences, are essential for improving long-term outcomes in affected populations.

## Introduction

### Background

The World Health Organization (WHO) estimates that approximately 1.3 billion individuals globally experience some form of disability [1]. Between 2016 and 2021, the prevalence of disability among noninstitutionalized adults in the United States increased from 24% to over 25% [2], driven by an aging population and enhanced disclosure of disability status following advances in societal acceptance and legal protections [3]. Findings from the Cognitive Function and Aging Study spanning from 1991 to 2011 indicated an increase in both total life expectancy and disability-free life expectancy; however, the proportion of life spent without disability has decreased [4]. Population aging in high-income countries is projected to persist, but evidence regarding trends in morbidity and disability prevalence remains inconclusive, with studies in the United States and Europe reporting increases, decreases, or stability in disability prevalence over time [5-8]. These disparities may be attributed to variations in the types of impairments and the prevalence of risk factors, including advanced age, unsafe living conditions, exposure to violence, malnutrition, poverty, and unhealthy behaviors [9,10]. Previous research has consistently reported health disadvantages among individuals who are disabled relative to their counterparts who are not disabled [11-13], as well as an elevated risk of mortality among those with disabilities [14,15].

Existing literature indicates that disability prevalence is significantly higher in older populations and often considered inevitable at the end of life [16,17]. Individuals who are disabled are known to have shorter lifespans compared to the general population [18,19]. Nevertheless, most studies rely on cross-sectional assessments of disability, lacking information on subsequent mortality risk. Longitudinal investigations are limited and tend to focus on incident disability rather than the longitudinal progression of disability after onset, often due to constraints in statistical power [20]. Both age at disability onset and the duration of disability can influence health outcomes [21]. While extensive research has been conducted on disability onset, the mortality risk associated with the duration of disability remains underexplored, and current findings regarding the mortality implications of disability onset age are inconsistent. On the one hand, emerging evidence suggests that early-onset disability is associated with a significantly higher risk of all-cause mortality compared to disability that develops later in life [22]. Individuals who become disabled at younger ages often experience prolonged exposure to functional limitations, comorbidities, and social disadvantages, which can accelerate physiological decline and increase the risk of death [17,23-25]. On the other hand, some studies have proposed that early-onset disability may allow more time for psychological adaptation and the development of coping strategies, which could potentially improve self-perceived health outcomes [26-28]. Despite these contrasting perspectives, most existing research estimates health outcomes based primarily on prevalent disability age, without fully accounting for the duration or severity of disability at onset, which may play a crucial role in shaping long-term survival.

### Objective

The objective of this study is to assess the all-cause mortality risk among individuals with new-onset activities of daily living (ADLs) or instrumental activities of daily living (IADLs) disabilities, compared to a control population, stratified by age at onset and disability severity in a representative national cohort.

## Methods

### Study Participants

This study used data from waves 10 to 16 (2010‐2023) of the Health and Retirement Study (HRS), a comprehensive longitudinal survey that examines the aging experiences of Americans aged ≥51 years. The HRS uses a multistage probability sampling design to yield a nationally representative sample of US adults in this age group [29]. Attrition rates varied from 10% to 19% across waves (Table S1 in Multimedia Appendix 1). The study collected self-reported data on a range of demographic, health, and functional indicators, including chronic health conditions, daily activities, disability status, and other determinants of health, at baseline and biennially thereafter. In 2006, the HRS expanded its methodology to include enhanced in-person interviews, which incorporated physical performance assessments, biomarker collections, and a psychosocial leave-behind questionnaire. A random half of the participants underwent these enhanced assessments in 2006, while the remaining participants were assessed in 2008, with this process continuing in subsequent waves. Detailed descriptions of the HRS’s recruitment methods and design have been published elsewhere [29].

For the ADL disability analysis, we included 28,062 participants from waves 10 to 16. We excluded 10,198 individuals for the following reasons: 2253 (22.09%) who did not attend the examination and already had a disability, 2709 (26.56%) with missing ADL information, and 5236 (51.34%) who were diagnosed with ADL disability at the time of interview. This left 17,864 eligible participants, among whom 5076 (28.41%) developed new-onset ADL disability during a median follow-up of 3.46 years (interquartile range [IQR], 0.54 - 4.88 years). Participants free of ADL disability at baseline were 1:1 matched with those who developed new-onset ADL disability, based on age (±1 y) and sex. After further excluding 1152 participants with missing data on key covariates, the final analytic sample for ADL disability consisted of 9000 individuals (4500 matched case-control pairs).

For the IADL disability analysis, 21,381 participants remained after excluding 6681 individuals for similar reasons (nonattendance with disability, missing IADL information, or IADL disability at interview). Among these 21,381 participants, 4759 (22.26%) developed new-onset IADL disability over a median follow-up of 3.42 years (IQR, 0.54–4.88 years)**.** Following the same age- and sex-matching procedure and excluding 998 participants with missing covariate data, the final IADL analysis included 8520 participants (4260 matched case-control pairs). To minimize bias due to unequal follow-up time or delayed outcome ascertainment, we implemented a matched cohort design. For each case identified as developing disability in a specific year, a control participant of similar age (+1 or –1 y) and sex, who was confirmed to be free of disability in the same wave, was selected. Follow-up for both the case and matched control commenced in that same year of disability onset for the case. For example, if a male participant aged 51 years developed ADL disability in 2010, a control was selected from male participants aged between 49 and 51 years who were disability-free at that time, and both were followed starting in 2010.

### Assessment of New-Onset Disability

In this study, disability was operationally defined as the participant’s inability to independently perform essential basic ADLs and IADLs. ADLs were measured using the Katz Index, which evaluates fundamental physical tasks such as dressing, bathing, feeding, transferring from bed to chair, toileting, and maintaining continence [30]. IADLs were assessed based on the Lawton scale, encompassing more complex tasks necessary for independent living, including preparing hot meals, medication management, financial management, grocery shopping, and telephone use [31]. Since wave 10 of the HRS, standardized questionnaires have systematically collected disability data by asking participants whether they experience difficulty performing these activities due to physical, mental, emotional, or memory problems, excluding any difficulties expected to last less than 3 months. Responses were coded dichotomously as *yes* (1) or *no* (0). Composite scores for ADLs and IADLs were calculated by summing individual item responses, resulting in a range from 0 to 5, where higher scores indicated greater difficulty severity. For both ADLs and IADLs, a score of ≥2 indicated severe difficulty, a score of 0 to 2 denoted mild difficulty, and any score >0 signified the presence of challenges in performing ADLs or IADLs. A score of 0 represented no reported difficulties.

### Outcomes

Mortality outcomes, including the year and month of death, were ascertained through exit interviews or via core interviews with the spouse or partner of the deceased participant.

### Data Collection

Sociodemographic characteristics, lifestyle factors (smoking status and physical activity), and medical history (including stroke, heart disease, and depressive symptoms) were collected via face-to-face interviews using a standardized survey protocol. Data collection occurred biennially, as previously described in the study by Fisher and Ryan [32]. Sociodemographic variables encompassed age (in years), sex (male or female), educational attainment (categorized as primary school or below, high school or equivalent, and college or above), and marital status (married vs unmarried). Current smoking status was determined using the question, “Do you currently smoke cigarettes?” Physical activity levels were classified into tertiles based on the International Physical Activity Questionnaire [33]. Depressive symptoms were assessed using the 8-item Center for Epidemiologic Studies Depression Scale, with the presence of 4 or more symptoms indicative of depression [34]. Heart disease and stroke diagnoses were confirmed by a physician during in-person evaluations using a structured questionnaire administered by the study personnel.

### Statistical Analysis

Study participants, comprising case participants with newly developed disabilities and their matched controls, were stratified into 4 onset age groups: <55 years, 55‐65 years, 65‐75 years, and ≥75 years. Continuous variables were presented as mean (SD), while categorical variables were expressed as percentages. Baseline characteristics between the case and control groups were compared using the Student *t* test (2-tailed) for continuous variables and the chi-square test for categorical variables. A trend analysis for individual factors across disability onset age groups was conducted by assigning the median value of each age group as a continuous variable within a separate model. General linear models were used for continuous variables, and logistic regression models were applied to categorical variables. The *P* values for trends were derived using the Wald test.

To examine the effect of disability onset on all-cause mortality across age groups, Cox proportional hazards regression analysis was used. The observation period spanned from the index date—the date of incident disability for the case (and the same time point for the matched control)—to either the date of death or the end of the follow-up period. Adjusted hazard ratios (AHRs) with 95% CIs for all-cause mortality were computed for participants with new-onset disabilities relative to their matched controls within each age group. All models were adjusted for sociodemographic variables, lifestyle factors, and relevant medical histories.

All survival analyses accounted for the complex survey design of the HRS, including sample weights, stratification, and clustering. To ensure nationally representative estimates and accurate variance estimation, wave-specific respondent-level weights were applied based on participants’ timing of entry into the study. Analyses were adjusted for the survey’s multistage sampling structure using appropriate design variables, and models were fitted using survey-weighted Cox proportional hazards regression with Taylor series linearization to estimate robust SEs.

Sensitivity analyses were conducted to validate the robustness of the findings. First, we excluded outcome events occurring within the initial 1 or 2 years of follow-up to mitigate potential reverse causation. Second, participants diagnosed with cancer by a physician or receiving active treatment for cancer were excluded to control for the confounding effects of severe cancer on mortality rather than disability. Third, we excluded participants with less than 2 years of follow-up to address potential biases introduced by short observation periods and ensure sufficient time for outcome ascertainment. Fourth, we performed E-value analyses to evaluate the potential impact of unmeasured confounding. Finally, we included participants with missing covariate data by implementing a 2-level multiple imputation by chained equations method under the assumption of missing at random. Statistical significance was defined as a *P* value of <.05. All statistical analyses were performed using Stata (version 17; StatCorp).

### Ethical Considerations

The HRS was approved by the institutional review boards of the University of Michigan and the National Institute on Aging. All participants provided written informed consent before data collection. The study procedures were conducted in accordance with the ethical standards of the responsible committees on human experimentation at both institutions, as well as with the principles of the Declaration of Helsinki. Participant privacy and confidentiality were strictly maintained throughout the study.

## Results

### Baseline Characteristics

**Figure 1.**
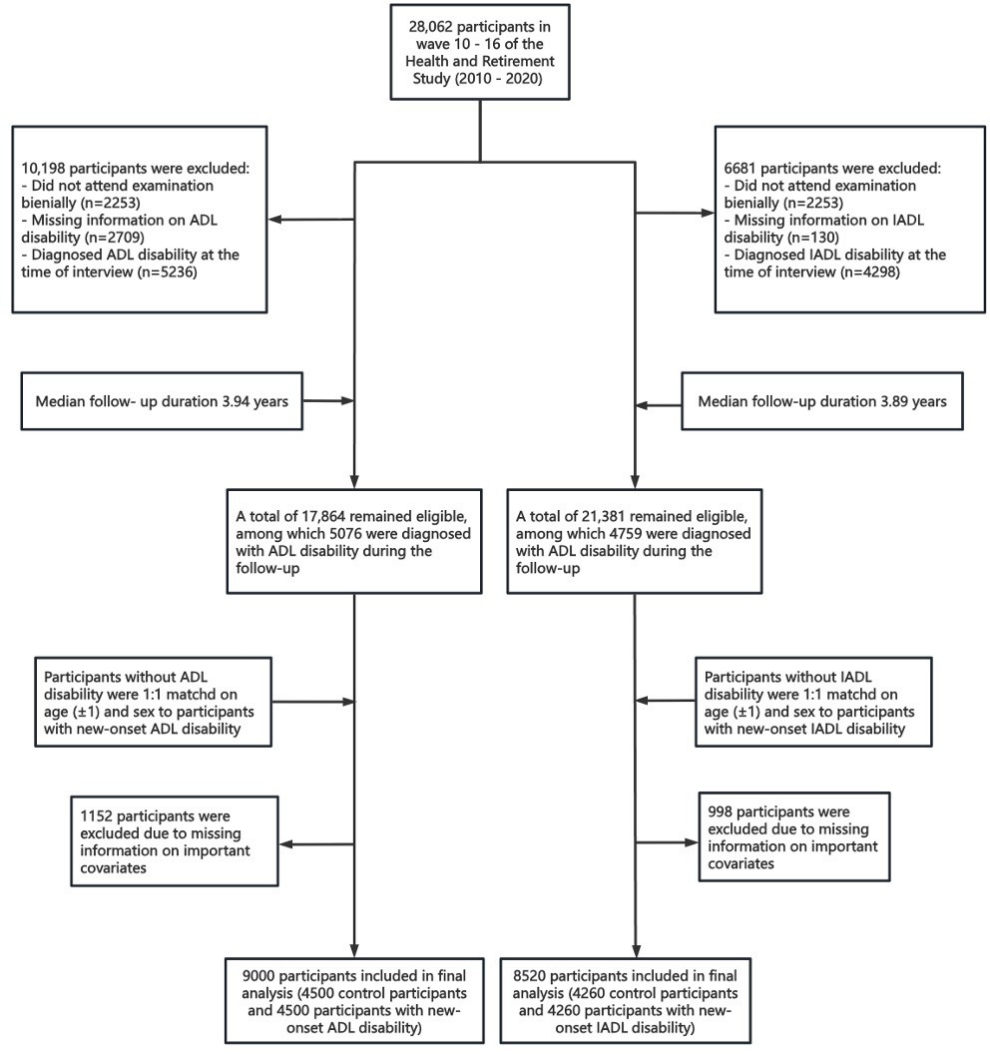
Study flowchart of participant selection. ADL: activity of daily living; IADL: instrumental activities of daily living.

Detailed participant screening and enrollment procedures are presented in Figure 1. Baseline characteristics of the study population are summarized in Tables1  2. The ADL analysis included a total of 9000 participants, among whom 4500 (50%) developed ADL disability during the follow-up period. Similarly, the IADL analysis comprised 8520 participants, with 4260 (51.64%) experiencing IADL disability during follow-up. The mean age at onset of ADL and IADL disability was 71.08 (SD 11.68) years and 71.74 (SD 12.09) years, respectively. Compared to control participants, individuals with new-onset ADL or IADL disability had lower levels of educational attainment, lower rates of marriage, a higher prevalence of current smoking, and a greater tendency to engage in light physical activity. In addition, a higher prevalence of cardiovascular disease (including heart disease and stroke) and depressive symptoms was observed in participants with new-onset disabilities (Table 1). Among those with younger-onset ADL or IADL disability, the likelihood of being a current smoker, experiencing depressive symptoms, and engaging in vigorous physical activity was higher relative to those with an older onset (Table 1).

**Table 1.** Baseline characteristics of participants included in the analysis of new-onset activity of daily living (ADL) disability.

	New-onset ADL disability	Control participants	*P* value	ADL disability onset age (y)				*P* value for trend
				<55	55‐65	65‐75	≥75	
Participants, n	4500	4500	—^a^	313	1239	967	1982	—
Age (y), mean (SD)	71.08 (11.68)	71.44 (11.36)	.51	50.74 (4.25)	59.55 (2.77)	69.51 (2.92)	82.26 (4.85)	<.001
Sex (male), n (%)	1788 (39.72)	1788 (39.74)	.99	100 (31.95)	505 (40.76)	388 (40.12)	795 (40.11)	.11
Educational background, n (%)			<.001					.14
Illiterate	1145 (25.44)	694 (15.43)		89 (28.43)	289 (23.33)	256 (26.47)	511 (25.78)	
Primary school or above	2538 (56.39)	2601 (57.81)		179 (57.19)	748 (60.37)	529 (54.71)	1082 (54.59)	
Secondary school or above	818 (18.17)	1204 (26.76)		45 (14.38)	202 (16.3)	182 (18.82)	389 (19.63)	
Married, n (%)	2093 (46.5)	2672 (59.39)	<.001	177 (56.55)	557 (44.96)	520 (53.77)	839 (42.33)	<.001
Current smoker, n (%)	691 (15.35)	447 (9.94)	<.001	112 (35.78)	323 (26.07)	148 (15.31)	108 (5.45)	<.001
Physical exercise, n (%)			<.001					<.001
Light	2178 (48.39)	1058 (23.52)		110 (35.14)	469 (37.85)	424 (43.85)	1175 (59.28)	
Moderate	1336 (29.68)	1510 (33.56)		97 (30.99)	437 (35.27)	309 (31.95)	493 (24.87)	
Vigorous	987 (21.93)	1931 (42.92)		106 (33.87)	333 (26.88)	234 (24.2)	314 (15.84)	
History of heart disease, n (%)	1539 (34.19)	1151(25.58)	<.001	64 (20.45)	270 (21.79)	339 (35.06)	866 (43.69)	<.001
History of stroke, n (%)	645 (14.33)	276 (6.13)	<.001	27 (8.63)	120 (9.69)	150 (15.51)	348 (17.56)	<.001
Depressive symptoms, n (%)	657 (14.6)	237 (5.27)	<.001	87 (27.8)	303 (24.46)	128 (13.24)	139 (7.01)	<.001

a Not available.

**Table 2.** Baseline characteristics of participants included in analysis of new-onset instrumental activity of daily living (IADL) disability.

	New-onset IADL disability	Control participants	*P* value	IADL disability onset age (y)				*P* value for trends
				<55	55-65	65-75	≥75	
Participants, n	4260	4260	—^a^	876	962	1036	1377	—
Age (y), mean (SD)	71.74 (12.09)	72.23 (11.75)	.03	54.92 (4.21)	64.10 (2.88)	75.03 (2.82)	85.30 (3.94)	<.001
Sex (male), n (%)	1724 (40.56)	1712 (40.1)	.67	330 (37.67)	398 (41.37)	427 (41.22)	569 (41.32)	.13
Educational background, n (%)			001					.06
Illiterate	1165 (27.41)	630 (14.76)		227 (25.91)	282 (29.31)	296 (28.57)	360 (26.14)	
Primary school or above	2377 (55.92)	2393 (56.06)		512 (58.45)	533 (55.41)	590 (56.95)	742 (53.89)	
Secondary school or above	709 (16.68)	1246 (29.29)		137 (15.64)	147 (15.28)	150 (14.48)	275 (19.97)	
Married, n (%)	1918 (45.12)	2502 (58.61)	<.001	396 (45.21)	459 (47.71)	538 (51.93)	525 (38.13)	<.001
Current smoker, n (%)	670 (15.76)	371 (8.69)	<.001	292 (33.33)	204 (21.21)	124 (11.97)	50 (3.63)	<.001
Physical exercise, n (%)			<.001					<.001
Light	2185 (51.4)	1066 (24.97)		332 (37.90)	448 (46.57)	556 (53.67)	849 (61.66)	
Moderate	1264 (29.73)	1322 (30.97)		293 (33.45)	315 (32.74)	289 (27.9)	367 (26.65)	
Vigorous	802 (18.87)	1881 (44.06)		251 (28.65)	199 (20.69)	191 (18.44)	161 (11.69)	
History of heart disease, n (%)	1523 (35.83)	1110 (26)	<.001	159 (18.15)	315 (32.74)	435 (41.99)	614 (44.59)	<.001
History of stroke, n (%)	668 (15.71)	273 (6.39)	<.001	78 (8.90)	130 (13.51)	200 (19.31)	260 (18.88)	<.001
Depressive symptoms, n (%)	651 (15.31)	193 (4.52)	<.001	259 (29.57)	186 (19.33)	114 (11)	92 (6.68)	<.001

a Not available.

### Associations Between Disability Onset Age and the Risk of Mortality

**Figure 2.**
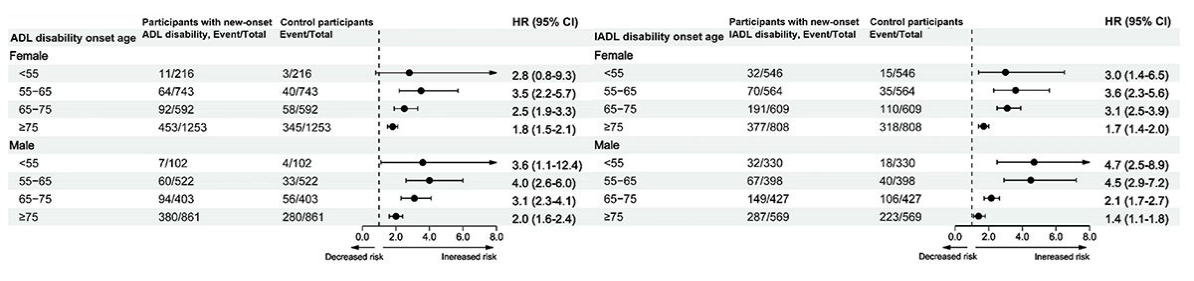
Adjusted hazard ratios (HRs) with 95% CIs for all-cause mortality in patients with new-onset activity of daily living (ADL) disability compared to control participants, stratified by age group and disability severity at onset. All models were adjusted for sociodemographic characteristics (age, sex, educational attainment, and marital status); lifestyle factors (smoking status and physical activity); and medical history (including stroke, heart disease, and depressive symptoms). IADL: instrumental activities of daily living.

**Figure 3.**
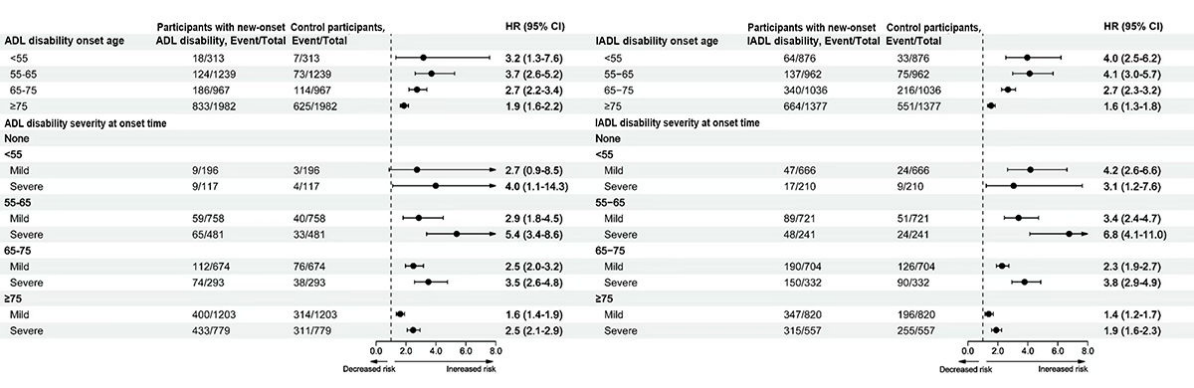
Adjusted hazard ratios (HRs) with 95% CIs for all-cause mortality in patients with new-onset activity of daily living (ADL) disability compared to controls, stratified by age group and gender. All models were adjusted for sociodemographic characteristics (age, sex, educational attainment, and marital status); lifestyle factors (smoking status and physical activity); and medical history (including stroke, heart disease, and depressive symptoms). IADL: instrumental activities of daily living.

Moreover, during the follow-up period, 25.79% (1161/4500) of participants with new-onset ADL disability and 28.35% (1208/4260) of those with new-onset IADL disability died, compared with 18.2% (819/4500) and 20.58% (877/4260) of their corresponding controls. After adjusting for key sociodemographic variables (age, sex, educational level, and marital status), lifestyle factors (smoking status and physical activity), and medical history (stroke, heart disease, and depressive symptoms), individuals with new-onset ADL or IADL disability were found to have a significantly increased risk of all-cause mortality compared to their matched controls across all age groups. Notably, participants with IADL disability onset between the ages of 55 and 65 years demonstrated the highest relative risk of all-cause mortality, with an AHR of 4.12 (95% CI 2.99‐5.68). In addition, this elevated risk was more pronounced among male participants (AHR 4.52, 95% CI 2.82‐7.17) compared to female participants (AHR 3.61, 95% CI 2.33‐5.60). The incremental risks gradually decreased with each 10-year increase of onset age. Similarly, participants with ADL disability onset between ages 55 and 65 years exhibited the highest relative risks, with an AHR of 5.38 (95% CI 3.37‐8.58) for severe IADL disability onset between the ages of 55 and 65 years. The relative risk of mortality also progressively declined with increasing age at disability onset (Figure 2), and male participants generally demonstrated higher risks than female participants across most age groups (Figure 3). We also graphically illustrated the incidence rates of all-cause mortality in different age groups in Figure S1 in Multimedia Appendix 1.

### Associations Between Disability Onset Severity and Risk of Mortality

Compared to participants with new-onset mild disability, those with new-onset severe ADL or IADL disability consistently exhibited higher relative risks across all age groups (Figure 2) and in the overall study population (Table S2 in Multimedia Appendix 1). For each onset age group, the AHRs for severe disability onset were consistently higher than for mild disability onset. However, the AHR for severe IADL disability onset below the age of 55 years was 3.05 (95% CI 1.22‐7.62), which was lower than that for mild IADL disability onset (AHR 4.17, 95% CI 2.64‐6.6). The incidence rates of all-cause mortality across various age groups, stratified by the severity of disability onset, are illustrated in Figure S2 in Multimedia Appendix 1.

### Sensitivity Analyses

Sensitivity analyses demonstrated that the association between new-onset disability and the risk of all-cause mortality remained robust across all age groups. This consistency persisted even after excluding participants who experienced outcome events within the first or second year of follow-up (Tables S3-S5 in Multimedia Appendix 1). Furthermore, the results remained unchanged upon excluding individuals with a history of cancer or those currently receiving treatment (Tables S3-S5 in Multimedia Appendix 1).

## Discussion

### Principal Findings

In this study, we observed that female participants diagnosed with ADL disability before the age of 55 years exhibited the highest relative risks for all-cause mortality when compared to age- and sex-matched controls. Participants with severe ADL or IADL disability at the time of diagnosis demonstrated greater mortality risks across all age groups compared to those with mild disability. Notably, the relative risk of mortality declined as the age of disability onset increased.

Previous literature has consistently identified disability as an independent predictor of mortality, even after adjusting for confounding factors such as cardiovascular disease [35], depressive symptoms [36], physical activity [37], and socioeconomic status [38]. The association between mortality and disability appears to be particularly pronounced for individuals with severe disabilities [39]. Infurna and Wiest [40] reported that the severity of disability plays a critical role in determining life satisfaction after onset, potentially due to the maintenance of an active lifestyle (eg, engagement in hobbies) or increased dependency on others, leading to a loss of independence. However, these studies primarily focused on participants with prevalent disabilities and did not initiate follow-up at the time of disability diagnosis. Although the precise mechanisms underlying the elevated mortality risk among individuals with disabilities remain unclear, our findings are biologically plausible. The relationship between disability and increased mortality risk in older adults is multifactorial, with age-related frailty being a key factor influencing both physical disability and mortality [41-43]. Decreases in muscle strength [42], chronic inflammation [44], neurological disorders [45], and chronic conditions [46] are interrelated factors that contribute to disability and concurrently accelerate mortality risk [42]. Multimorbidity is also closely associated with increased risks of both disability and mortality in older populations [47]. In addition, physical frailty and loss of independence often complicate the management of chronic conditions, leading to worse health outcomes. [48]

Our findings underscore the importance of early preventive interventions for individuals who experience disability onset before the age of 65 years, particularly those with severe functional impairments. Although disability onset has been extensively examined, research on how age at onset and severity influence mortality risk remains limited. Similar to our results, a population-based cohort study with over 10 years of follow-up [49] found that younger individuals with ADL impairments experience a significant reduction in survival. Only 2 previous studies have explicitly considered age as a timescale when assessing mortality risk [39,50]. Lamarca et al [50] demonstrated that earlier onset of ADL disability was associated with a higher mortality risk compared to older adults, with women less likely to recover from disability compared to men. Majer et al [39] similarly found that the mortality risk associated with disability decreased with increasing age, suggesting that younger individuals are more adversely affected by disability in terms of mortality risk. Nonetheless, some studies have suggested that younger onset of disability has a less detrimental effect on mortality in older populations, a finding that contrasts with our results [40,51,52]. These discrepancies may be attributable to differences in age stratification, disability classification [53,54], and the duration of postdiagnosis care [55] across studies.

The early identification of disability is of critical importance, as it has been consistently linked to an elevated risk of mortality. Individuals who experience disability onset at younger ages, such as those aged <55 years or aged between 55 and 65 years, demonstrate a heightened risk of mortality compared to those with onset between 65 and 75 years or those aged >75 years. This disparity in risk can be attributed to multiple factors. First, younger individuals with disabilities typically endure prolonged durations of illness and its associated complications, contributing to increased mortality rates [56]. In addition, the earlier onset of disability may signal the presence of more aggressive underlying pathological processes, thereby influencing adverse outcomes. Furthermore, disability at a younger age may significantly affect quality of life and psychosocial well-being, potentially resulting in detrimental health behaviors or insufficient social support, both of which compound health risks. Our findings also indicate that among individuals of the same gender, those aged <65 years with IADL disability exhibit a higher mortality risk than those with ADL impairment. In contrast, in populations aged ≥65 years, the pattern reverses: individuals with ADL disability face a greater mortality risk than those with IADL limitations. This crossover effect may reflect the different implications of functional limitations at various life stages. In middle-aged adults, IADL disability could be an early marker of accelerated biological aging or cognitive decline, often preceding ADL limitations and signaling broader systemic deterioration [57,58]. These individuals may still appear physically capable but experience subtle impairments in executive functioning or complex task management, which have been linked to excess mortality [59]. However, in older adults, ADL impairment reflects more severe physical frailty and dependency, often closely associated with terminal decline and higher short-term mortality risk. [60,61]

Our study further elucidates gender-specific differences in the association between disability onset and all-cause mortality. Specifically, we observed stronger mortality risks among men than women, corroborating existing literature [39,62-64] and aligning with the established gender paradox in morbidity and mortality [65,66]. Women tend to experience more frequent and prolonged periods of disability, which may contribute to their lower recovery and mortality rates relative to men [67,68]. Conversely, men are more susceptible to fatal conditions such as cardiovascular disease and stroke, which elevates their mortality risk [69]. This observed gender disparity in disability and mortality may not be completely explained by gender-specific health conditions, body composition, or the operational definitions of disability [70]. Therefore, additional investigation into alternative explanatory pathways is warranted.

This study offers several notable strengths. It is the first large-scale prospective study to examine the impact of disability onset age on all-cause mortality, with participants tracked from the time of diagnosis. Moreover, the inclusion of age- and sex-matched controls without disability allows for a comprehensive consideration of potential confounders. We also accounted for the severity of disability at onset, distinguishing between mild and severe cases, thereby capturing the influence of both duration and severity on mortality outcomes. Furthermore, repeated clinical examinations provided access to a wide range of covariates, enabling the minimization of confounding bias.

### Limitations

Despite the strengths, several limitations must be acknowledged. First, the onset age of disability was approximated based on the date of initial investigation minus the participant’s birthdate, which may differ slightly from the precise diagnosis age. Nevertheless, given that participants were interviewed biennially, such discrepancies are unlikely to substantially impact the findings. Second, although adjustments were made for a range of factors, residual confounding cannot be entirely ruled out, especially from unmeasured factors such as nutritional status or exposure to violence. However, our E-value analysis provides quantitative reassurance regarding the validity of our findings (Table S6 in Multimedia Appendix 1). Third, disability status and severity were based on self-report, which may be subject to recall or interpretation biases. Notably, the HRS explicitly instructs respondents to exclude any difficulties expected to last fewer than 3 months when reporting activity limitations. This reduces the risk of misclassifying temporary impairments, such as those due to acute illness or injury, as new-onset disability. Moreover, while self-reported difficulty does not necessarily imply complete dependence, it has been shown to be a meaningful indicator of early functional decline in aging research. Fourth, because disability is a dynamic process with potential for recovery, our approach—which identifies incident disability at the first report of difficulty—may include cases that later resolve. However, requiring confirmation across multiple waves would risk underestimating meaningful early disability and may introduce survivor bias by excluding individuals who recover or die before a second measurement. Moreover, our use of a standardized and widely accepted definition of ADL or IADL disability, along with multiple sensitivity analyses, supports the robustness of our findings. Finally, we acknowledge the potential for survivor bias, as individuals who develop disability at older ages must have survived without severe illness to that point. However, this bias is mitigated by our use of age-at-onset stratification and incident (rather than prevalent) disability, which allows for fairer comparisons within age groups and reduces the overrepresentation of unusually resilient individuals.

### Conclusions

This study demonstrates that the relative risks of all-cause mortality vary significantly depending on the age at which disability is diagnosed, with a notably higher risk observed among individuals diagnosed at a younger age. The identification and precise quantification of the elevated mortality risk associated with early-onset disability provide critical insights for risk stratification at earlier stages of the condition. Such stratification may inform the development of targeted interventions, including intensive therapeutic approaches and tailored lifestyle modifications, aimed at mitigating the risk of complication-related morbidity and mortality in this growing population of individuals with early-onset disability.

## Supplementary material

10.2196/73254 Multimedia Appendix 1 Detailed incidence rates, attrition rates, main and sensitivity analyses, and robustness checks of the associations between disability onset, severity, and all-cause mortality among older adults in the Health and Retirement Study.

## Abbreviations

ADL: activity of daily living
AHR: adjusted hazard ratio
HR: hazard ratio
HRS: Health and Retirement Study
IADL: instrumental activity of daily living
WHO: World Health Organization
