# Supplementary material for: Association Between Age and Severity at Disability Onset and All-Cause Mortality: Longitudinal Observational Study From the Health and Retirement Study
Source: JMIR Aging. 2025 Oct 3;8:e73254. doi: 10.2196/73254 (PMC12494357; doi:10.2196/73254)
Supplement: Multimedia Appendix 1 [file aging-v8-e73254-s001.docx]

**Supplementary Information to “Association Between Age and Severity at Disability Onset and All-cause Mortality: The Health and Retirement Study”**

**Figure S1.** Incidence Rates of All-Cause Mortality in Participants with New-Onset ADL Disability Compared to Controls, Stratified by Onset Age Groups.

**Figure S2.** Incidence Rates of All-Cause Mortality in Participants with New-Onset ADL Disability Compared to Controls, Stratified by Onset Age Groups and Disability Severity.

**Table S1.** Attrition Rates (Mortality or Loss to follow up) of each wave in Health and Retirement Study.

**Table S2.** Association between ADL disability severity at onset time and all-cause mortality among older adults.

**Table S3.** Sensitivity analysis of the association between disability onset age and all-cause mortality among older adults.

**Table S4.** Sensitivity analysis of the association between disability severity and all-cause mortality among older adults.

**Table S5.** Sensitivity analysis of the association between disability severity by onset age groups and all-cause mortality among older adults.

**Table S6.** Associations Between Disability and All-Cause Mortality: Results from Survey-Weighted Cox Regression Models with E-values

**Figure S1. Incidence Rates of All-Cause Mortality in Participants with New-Onset Disability Compared to Controls, Stratified by Onset Age Groups.**

**Figure S2. Incidence Rates of All-Cause Mortality in Participants with New-Onset Disability Compared to Controls, Stratified by Onset Age Groups and Disability Severity.**

*Notes: Within each age group, the left two bars represent participants with mild disability at onset, while the right two bars represent those with severe disability at onset.*

**Table S1. Attrition Rates (Mortality or Loss to follow up) of each wave in Health and Retirement Study.**

| **Year of Waves** | **HRS** | |
| --- | --- | --- |
|  | **N** | **Attrition Rate (%)** |
| **2010** | 22034 |  |
| **2011** |  |  |
| **2012** | 19781 | 10.23 |
| **2013** |  |  |
| **2014** | 17509 | 11.49 |
| **2015** |  |  |
| **2016** | 15070 | 13.93 |
| **2017** |  |  |
| **2018** | 12167 | 19.26 |
| **2019** |  |  |

**Table S2. Association between disability severity at onset time and all-cause mortality among older adults.**

|  | HR (95% CI) | P Value |  | HR (95% CI) | P Value |
| --- | --- | --- | --- | --- | --- |
| ADL Disability Severity  at Onset Time | | | IADL Disability Severity  at Onset Time | | |
| None |  |  | None |  |  |
| Mild | 1.75 (1.55 - 1.98) | <.001 | Mild | 1.82 (1.56 - 2.11) | <.001 |
| Severe | 2.8 (2.45 - 3.2) | <.001 | Severe | 2.63 (2.26 - 3.08) | <.001 |

**Table S3. Sensitivity analysis of the association between disability onset age and all-cause mortality among older adults.**

| **ADL Disability Onset Age** | Sensitivity I  (N = 8, 990) | Sensitivity II  (N = 8,923) | Sensitivity III  (N = 6, 985) | Sensitivity IV  (N = 6, 561) | Sensitivity V  (N = 9, 369) |
| --- | --- | --- | --- | --- | --- |
|  | HR (95% CI), P |  |  |  |  |
| <55 | 3.12 (1.85 – 5.27), <.001 | 3.09 (1.80 – 5.29), <.001 | 3.59 (1.97 – 6.56), <.001 | 3.52 (1.09 – 11.29), 0.035 | 3.31 (1.40–7.85), .007 |
| 55–65 | 2.80 (2.16 – 3.63), <.001 | 2.83 (2.18 – 3.69), <.001 | 3.06 (2.26 – 4.14), <.001 | 3.90 (2.54 – 5.99), <.001 | 3.94 (2.77–5.62), <.001 |
| 65–75 | 2.39 (1.97 – 2.89), <.001 | 2.40 (1.98 – 2.92), <.001 | 2.41 (1.91 – 3.05), <.001 | 2.76 (2.07 – 3.68), <.001 | 2.79 (2.23–3.49), <.001 |
| ≥75 | 1.95 (1.72 – 2.20), <.001 | 1.93 (1.70 – 2.18), <.001 | 1.98 (1.71 – 2.30), <.001 | 1.98 (1.67 – 2.35), <.001 | 1.89 (1.64–2.19), <.001 |
| **IADL Disability Onset Age** | Sensitivity I  (N = 8, 507) | Sensitivity II  (N = 8, 429) | Sensitivity III  (N = 6, 648) | Sensitivity IV  (N = 5,782) | Sensitivity V  (N = 8, 901) |
|  | HR (95% CI), P |  |  |  |  |
| <55 | 2.83 (2.01 - 3.99), <.001 | 2.79 (1.96 - 3.96), <.001 | 2.99 (2.04 - 4.39), <.001 | 6.21 (3.36 - 11.49), <.001 | 1.08 (0.43 – 2.75), 0.865 |
| 55–65 | 2.47 (1.94 - 3.14), <.001 | 2.46 (1.92 - 3.16), <.001 | 2.35 (1.77 - 3.12), <.001 | 3.84 (2.36 - 6.27), <.001 | 3.42 (2.48 – 4.73), <0.001 |
| 65–75 | 2.31 (1.99 - 2.69), <.001 | 2.31 (1.98 - 2.69), <.001 | 2.24 (1.87 - 2.68), <.001 | 2.78 (2.19 - 3.54), <.001 | 3.28 (2.59 – 4.15), <0.001 |
| ≥75 | 1.56 (1.37 - 1.76), <.001 | 1.59 (1.40 - 1.81), <.001 | 1.59 (1.36 - 1.85), <.001 | 1.91 (1.55 - 2.35), <.001 | 1.74 (1.52 – 1.99), <0.001 |

Notes:

*Sensitivity I: Exclude death occurred in the first year of follow-up; Sensitivity II: Exclude death occurred in the first two year of follow-up; Sensitivity III: Exclude participants who were diagnosed with cancer by physician or being on treatment; Sensitivity IV: Exclude participants with a follow-up time of < 2 years.*

**Table S4. Sensitivity analysis of the association between disability severity and all-cause mortality among older adults.**

| ADL Disability Severity | Sensitivity I  (N = 8,990) | Sensitivity II  (N = 8,923) | Sensitivity III  (N = 6,985) | Sensitivity IV  (N = 6,561) | Sensitivity V  (N = 9, 369) |
| --- | --- | --- | --- | --- | --- |
|  | HR (95% CI), P |  |  |  |  |
| None | Reference | Reference | Reference | Reference | Reference |
| Mild | 1.75 (1.55 – 1.99), <.001 | 1.74 (1.54 - 1.98), <.001 | 1.82 (1.56 - 2.11), <.001 | 2.02 (1.567 - 2.46), <.001 | 1.91 (1.62 – 2.25), <.001 |
| Severe | 2.79 (2.44 – 3.19), <.001 | 2.79 (2.43 - 3.20), <.001 | 2.79 (2.38 - 3.28) <.001 | 3.01 (2.47 - 3.67), <.001 | 2.92 (2.49 – 3.42), <.001 |
| IADL Disability Severity | Sensitivity I  (N = 8,507) | Sensitivity II  (N = 8,429) | Sensitivity III  (N = 6,648) | Sensitivity IV  (N = 5,782) | Sensitivity V  (N = 8, 901) |
|  | HR (95% CI), P |  |  |  |  |
| None | Reference | Reference | Reference | Reference | Reference |
| Mild | 1.63 (1.45 - 1.83), <.001 | 1.65 (1.47 - 1.86), <.001 | 1.63 (1.42 - 1.88), <.001 | 2.29 (1.89 - 2.78), <.001 | 1.63 (1.45 - 1.83), <.001 |
| Severe | 2.27 (1.99 - 2.59), <.001 | 2.29 (2.00 - 2.62), <.001 | 2.30 (1.97 - 2.70), <.001 | 2.52 (2.08 - 3.06), <.001 | 2.33 (2.06 - 2.64), <.001 |

Notes: *All models were adjusted for age, education, sex, marital status, heart disease medication, stroke medication, physical activity, smoking, and depression. Sensitivity I: Exclude death occurred in the first year of follow-up; Sensitivity II: Exclude death occurred in the first two year of follow-up; Sensitivity III: Exclude participants who were diagnosed with cancer by physician or being on treatment; Sensitivity IV: Exclude participants with a follow-up time of < 2 years; Sensitivity V: All estimates derived from survey-weighted Cox proportional hazards models using multiple imputation (MI, 10 datasets).*

**Table S5. Sensitivity analysis of the association between disability severity by onset age groups and all-cause mortality among older adults.**

| **ADL Disability Severity**  **at Onset Age** | Sensitivity I  (N = 8, 990) | Sensitivity II  (N = 8,923) | Sensitivity III  (N = 6, 985) | Sensitivity IV  (N = 6, 561) | Sensitivity V  (N = 9, 369) |
| --- | --- | --- | --- | --- | --- |
|  | HR (95% CI), P |  |  |  |  |
| <55: Mild | 2.47 (1.23 – 4.95), 0.011 | 2.57 (1.28 – 5.18), 0.008 | 2.40 (1.02 – 5.62), 0.045 | 3.40 (0.70 – 16.58), 0.129 | 2.87 (0.95–8.65), .062 |
| <55: Severe | 4.08 (2.03 – 8.20), <.001 | 3.80 (1.81 – 7.94), <.001 | 5.44 (2.56 – 11.59), <.001 | 3.93 (0.73 – 21.33), 0.111 | 4.22 (1.16–15.27), .029 |
| 55–65: Mild | 2.30 (1.68 – 3.16), <.001 | 2.36 (1.71 – 3.25), <.001 | 2.68 (1.87 – 3.83), <.001 | 3.00 (1.60 – 5.65), 0.001 | 2.96 (1.87–4.62), <.001 |
| 55–65: Severe | 3.43 (2.51 – 4.69), <.001 | 3.44 (2.49 – 4.73), <.001 | 3.52 (2.43 – 5.10), <.001 | 5.84 (3.33 – 10.24), <.001 | 5.93 (3.67–9.57), <.001 |
| 65–75: Mild | 2.04 (1.63 – 2.55), <.001 | 2.08 (1.66 – 2.60), <.001 | 2.05 (1.56 – 2.70), <.001 | 2.63 (1.86 – 3.73), <.001 | 2.59 (2.03–3.33), <.001 |
| 65–75: Severe | 3.31 (2.53 – 4.31), <.001 | 3.29 (2.51 – 4.32), <.001 | 3.34 (2.42 – 4.61), <.001 | 3.35 (2.21 – 5.07), <.001 | 3.44 (2.52–4.71), <.001 |
| ≥75: Mild | 1.64 (1.43 – 1.88), <.001 | 1.61 (1.40 – 1.86), <.001 | 1.68 (1.42 – 1.99), <.001 | 1.77 (1.44 – 2.18), <.001 | 1.64 (1.39–1.94), <.001 |
| ≥75: Severe | 2.61 (2.25 – 3.03), <.001 | 2.62 (2.25 – 3.04), <.001 | 2.58 (2.16 – 3.09), <.001 | 2.59 (2.02 – 3.33), <.001 | 2.47 (2.08–2.94), <.001 |
| **IADL Disability Severity**  **at Onset Age** | Sensitivity I  (N = 8, 507) | Sensitivity II  (N = 8, 429) | Sensitivity III  (N = 6, 648) | Sensitivity IV  (N = 5,782) | Sensitivity V  (N = 8, 901) |
|  | HR (95% CI), P |  |  |  |  |
| <55: Mild | 2.86 (1.97 - 4.14), <.001 | 2.87 (1.96 - 4.19), <.001 | 3.11 (2.07 - 4.65), <.001 | 6.79 (3.66 - 12.59), <.001 | 0.91 (0.28 – 2.93), 0.873 |
| <55: Severe | 2.81 (1.62 - 4.86), <.001 | 2.60 (1.45 - 4.64), 0.001 | 2.57 (1.33 - 4.95), 0.005 | 3.35 (0.80 - 14.01), 0.096 | 1.73 (0.43 – 6.98), 0.435 |
| 55–65: Mild | 2.18 (1.66 - 2.85), <.001 | 2.21 (1.68 - 2.91), <.001 | 2.17 (1.59 - 2.95), <.001 | 3.85 (2.37 - 6.23), <.001 | 3.14 (2.18 – 4.87), <0.001 |
| 55–65: Severe | 3.48 (2.47 - 4.91), <.001 | 3.35 (2.34 - 4.79), <.001 | 3.00 (1.96 - 4.58), <.001 | 3.73 (1.60 - 8.72), 0.003 | 4.32 (2.43 – 7.69), <0.001 |
| 65–75: Mild | 1.94 (1.63 - 2.32), <.001 | 1.93 (1.61 - 2.31), <.001 | 1.90 (1.54 - 2.34), <.001 | 2.64 (2.02 - 3.46), <.001 | 2.93 (2.34 – 3.69), <0.001 |
| 65–75: Severe | 3.21 (2.63 - 3.93), <.001 | 3.24 (2.63 - 3.98), <.001 | 3.07 (2.41 - 3.91), <.001 | 3.21 (2.41 - 4.27), <.001 | 4.37 (2.96 – 6.27), <0.001 |
| ≥75: Mild | 1.40 (1.21 - 1.62), <.001 | 1.44 (1.24 - 1.67), <.001 | 1.37 (1.15 - 1.64), <.001 | 1.81 (1.41 - 2.32), <.001 | 1.52 (1.30 – 1.78), <0.001 |
| ≥75: Severe | 1.86 (1.60 - 2.18), <.001 | 1.90 (1.62 - 2.23), <.001 | 1.98 (1.64 - 2.38), <.001 | 2.16 (1.69 - 2.77), <.001 | 2.22 (1.91 – 2.58), <0.001 |

Notes:

*Sensitivity I: Exclude death occurred in the first year of follow-up; Sensitivity II: Exclude death occurred in the first two year of follow-up; Sensitivity III: Exclude participants who were diagnosed with cancer by physician or being on treatment; Sensitivity IV: Exclude participants with a follow-up time of < 2 year; Sensitivity V: All estimates derived from survey-weighted Cox proportional hazards models using multiple imputation (MI, 10 datasets).*

**Table S6. Associations Between Disability and All-Cause Mortality: Results from Survey-Weighted Cox Regression Models with E-values**

|  |  | \| **HR (95% CI)** \| \| --- \| | \| **E-value**  **(Point Estimate)** \| \| --- \| | \| **E-value**  **(Lower Bound)** \| \| --- \| |
| --- | --- | --- | --- | --- | --- | --- | --- |
| **ADL Disability Severity**  **at Onset Time** | Mild Disability | 1.86 (1.58–2.20) | 2.440 | 2.087 |
|  | Severe Disability | 2.89 (2.48–3.38) | 3.553 | 3.137 |
| **ADL Disability Onset Age** | <55 years | 3.16 (1.32–7.58) | 3.812 | 1.719 |
|  | 55–65 years | 3.70 (2.61–5.24) | 4.304 | 3.277 |
|  | 65–75 years | 2.73 (2.20–3.39) | 3.396 | 2.834 |
|  | ≥75 years | 1.86 (1.62–2.15) | 2.446 | 2.133 |
| **ADL Disability Severity**  **at Onset Age** | <55: Mild | 2.73 (0.88–8.46) | 3.397 | 1.000 |
|  | <55: Severe | 3.98 (1.11–14.33) | 4.545 | 1.352 |
|  | 55–65: Mild | 2.85 (1.81–4.48) | 3.513 | 2.382 |
|  | 55–65: Severe | 5.38 (3.37–8.58) | 5.640 | 4.005 |
|  | 65–75: Mild | 2.49 (1.96–3.16) | 3.143 | 2.555 |
|  | 65–75: Severe | 3.49 (2.56–4.76) | 4.117 | 3.223 |
|  | ≥75: Mild | 1.61 (1.36–1.90) | 2.124 | 1.779 |
|  | ≥75: Severe | 2.47 (2.08–2.93) | 3.126 | 2.699 |
| **IADL Disability Severity**  **at Onset Time** | Mild Disability | 1.82 (1.56–2.12) | 2.388 | 2.061 |
|  | Severe Disability | 2.63 (2.26–3.08) | 3.296 | 2.895 |
| **IADL Disability Onset Age** | <55 years | 3.97 (2.53–6.21) | 4.530 | 3.191 |
|  | 55–65 years | 4.12 (2.99–5.68) | 4.660 | 3.650 |
|  | 65–75 years | 2.67 (2.25–3.17) | 3.334 | 2.889 |
|  | ≥75 years | 1.55 (1.32–1.82) | 2.044 | 1.711 |
| **IADL Disability Severity**  **at Onset Age** | <55: Mild | 4.17 (2.64–6.60) | 4.702 | 3.298 |
|  | <55: Severe | 3.05 (1.22–7.62) | 3.710 | 1.566 |
|  | 55–65: Mild | 3.38 (2.43–4.71) | 4.020 | 3.087 |
|  | 55–65: Severe | 6.75 (4.13–11.04) | 6.588 | 4.665 |
|  | 65–75: Mild | 2.28 (1.90–2.73) | 2.918 | 2.490 |
|  | 65–75: Severe | 3.78 (2.93–4.86) | 4.367 | 3.594 |
|  | ≥75: Mild | 1.39 (1.15–1.69) | 1.828 | 1.435 |
|  | ≥75: Severe | 1.89 (1.58–2.25) | 2.472 | 2.083 |

*Notes: ADL = Activities of Daily Living; IADL = Instrumental Activities of Daily Living; HR = Hazard Ratio; CI = Confidence Interval；E-value (Point estimate) represents the minimum strength of association, on the risk ratio scale, that an unmeasured confounder would need to have with both the exposure and the outcome to fully explain away the observed association, conditional on the measured covariates; E-value (CI) corresponds to the minimum strength of association that an unmeasured confounder would need to have with both the exposure and the outcome to move the lower bound of the confidence interval to include the null.*
